# Establishing the Optimal Density of the Michell Truss Members

**DOI:** 10.3390/ma13173867

**Published:** 2020-09-01

**Authors:** Tomáš Stejskal, Miroslav Dovica, Jozef Svetlík, Peter Demeč, Lukáš Hrivniak, Michal Šašala

**Affiliations:** 1 Department of Manufacturing Machinery, Faculty of Mechanical Engineering, The Technical University of Kosice, Letná 9, 04200 Košice, Slovakia; tomas.stejskal@tuke.sk (T.S.); jozef.svetlik@tuke.sk (J.S.); peter.demec@tuke.sk (P.D.); lukas.hrivniak@tuke.sk (L.H.); 2 Department of Biomedical Engineering and Measurement, Faculty of Mechanical Engineering, The Technical University of Kosice, Letná 9, 04200 Košice, Slovakia; miroslav.dovica@tuke.sk

**Keywords:** Michell truss, golden section, topology optimization

## Abstract

Topology optimization is a dynamically developing area of industrial engineering. One of the optimization tasks is to create new part shapes, while maintaining the highest possible stiffness and reliability and minimizing weight. Thanks to computer technology and 3D printers, this path of development is becoming more and more topical. Two optimization conditions are often used in topology optimization. The first is to achieve the highest possible structure stiffness. The second is to reduce the total weight of the structure. These conditions do not have a direct effect on the number of elements in the resulting structure. This paper proposes a geometric method that modifies topological structures in terms of the number of truss elements but is not based on the optimization conditions. The method is based on natural patterns and further streamlines the optimization strategies used so far. The method’s efficiency is shown on an ideal Michell truss.

## 1. Introduction

The environment can be improved by saving materials. For example, in biomimetic systems, the area of noise and vibration diagnostics reduces failure rates and increases reliability [1]. It constitutes only one of the options, though. In this paper, the option of improving the design of machines and products is explored. Topology optimization allows for a significant weight reduction. Additive technologies are used to this end, which must be verified in practical tests [2]. Last but not least, all these new technologies are supported by modern mathematical philosophies, e.g., by similarity methods copying natural patterns [3].

In connection with natural patterns, it is often possible to encounter the principle of even distribution of stresses over the entire object after it is loaded with external forces. The description of this principle is called “Michell structures.” The foundations of the principle were laid by the now frequently cited paper on the optimization condition for the economical limits of material loss from 1904 by A.G.M. Michell [4]. The optimization principle is quite significant, but its real application did not occur until the early 1960s, with the development of computer technology.

**Figure 1:**
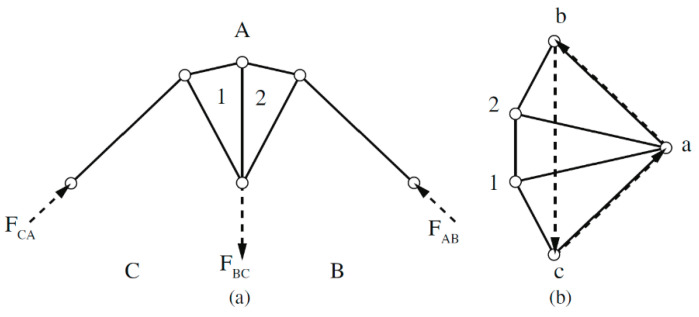
Example of reciprocal diagrams: (**a**) form diagram and (**b**) force diagram [6].

The principle itself is based on the deformation condition, which is given by Maxwell’s load-path theorem [5] (paper by J.C. Maxwell from 1864: “On Reciprocal Figures and Diagrams of Forces”). Based on the theorem, the load scheme of a truss can be converted to a force scheme and vice versa. The total length of the loaded members (form diagram) and the total length of the internal force vectors acting in the individual members (force diagram) can be read from the diagrams. With different truss structures, but with a given external load, the length of the components changes reciprocally with respect to the length of the force effects. Therefore, these schemes are called reciprocal (see Figure 1).

Maxwell’s theorem states that the sum of the tensile load paths in the structure minus the sum of the load paths in compression equals the value of work related to the external forces applied (reactions including). Speaking mathematically [5]

(1) $$\sum F_{T}L_{T}-\sum F_{C}L_{C}=\sum {\bar{P}}_{i}{\bar{r}}_{i}$$

The term “load path” means the product of the force and the length of the member F L. Here, $F_{T}$ are the forces in the members caused by the tension, $F_{C}$ are the forces in the members caused by compression, $L_{T}$ are the lengths of the members under tension, $L_{C}$ are the lengths of the members under compression, ${\bar{P}}_{i}$ is the *i*th external force, and ${\bar{r}}_{i}$ is the distance of the *i*th external force from the origin.

In different members’ arrangements, with the external loads unchanged, the value of the Equation (1) result changes. Changing the shape may result in a change in the magnitude of the force but, at the same time, the length of members changes, too. Thus, it can be assumed that there is such a combination of the shape of the structure and the lengths of members, at which a local minimum is achieved throughout the structure. This assumption leads to the first optimization condition expressed by Michell in his work.

The local minimum of Equation (1) is obtained under the following optimization condition (2) [7]:

“The minimum of Equation (1) is achieved when there is the same tension or compression in each member of the structure.”

(2) $$\sum {\bar{P}}_{i}{\bar{r}}_{i}=\min$$

For this ideal case, Michell created some cases of continuous structures in the continuum. An analytical solution is only possible for infinitely many infinitely short members. Fulfillment of the above condition presupposes a symmetrical structure with a symmetrical load on its nodes. Michell’s optimization statement verbally includes the second optimization condition that concerns weight savings. That statement is as follows [4]:

“A frame (today called truss) (is optimal) attains the limit of economy of material possible in any frame-structure under the same applied forces, if the space occupied by it can be subjected to an appropriate small deformation, such that the strains in all the bars of the frame are increased by equal fractions of their lengths, not less than the fractional change of length of any element of the space.”

The optimization condition (2) does not minimize the weight of the structure, it only allows it as an option. Based on Michell’s statement, the second optimization condition is defined in the professional literature as the aim to minimize the volume of matter (3), at which the permissible stress (4) must not be exceeded [5]. Weight minimization condition

(3) $$\underset{x}{\min}=\sum_{e}x^{\left(e\right)}L^{\left(e\right)}$$

Permissible stress interval

(4) $$-{\bar{\sigma }}_{C}\leq \sigma^{\left(e\right)}\leq {\bar{\sigma }}_{T}$$

where $x^{\left(e\right)}$, the cross-section of the element (member) is *e*, $L^{\left(e\right)}$ is the length of the element, $\sigma^{\left(e\right)}$ is the stress inside the element, ${\bar{\sigma }}_{C}$ is the permissible compression, and ${\bar{\sigma }}_{T}$ is the permissible tension.

Since a homogeneous mass is considered, the minimum cross-section of the elements essentially also determines the minimum weight of the structure. The indicated optimization of the truss weight is technically referred to as topology optimization. It is an optimization under conditions that do not require limiting the shape of the structure.

The issue of optimization focuses on finding the best solution among all possible solutions. It is, therefore, appropriate to define an ideal topology solution.

The ideal solution for an object under load is a shape of maximum stiffness in direction of the load, minimum weight, and the maximum permissible stress in each of its parts.

This ideal solution can be achieved by various optimization procedures. The objective function is defined theoretically, describing the material density distribution in a design space.

(5) $$F(u(\rho ),\rho )=\int_{\Omega }f(u(\rho ),\rho )dV$$

where F(u(ρ),ρ) is the objective function, Ω is design space, ρ is material density, and V is the object volume. The goal of optimization is to minimize the mass while maintaining the following constraint.

(6) $$G_{0}(u(\rho ),\rho )=\int_{\Omega }\rho dV-V_{0}\leq 0$$

where $G_{0}(u(\rho ),\rho )$ is a limit on the amount of material, or a limit on the maximum stress. Although the description of optimization is general, the solution it points to is the ideal solution, i.e., minimum weight, maximum stiffness, and full utilization of the material in terms of achieving the permissible stress in its entire volume.

**Figure 2:**
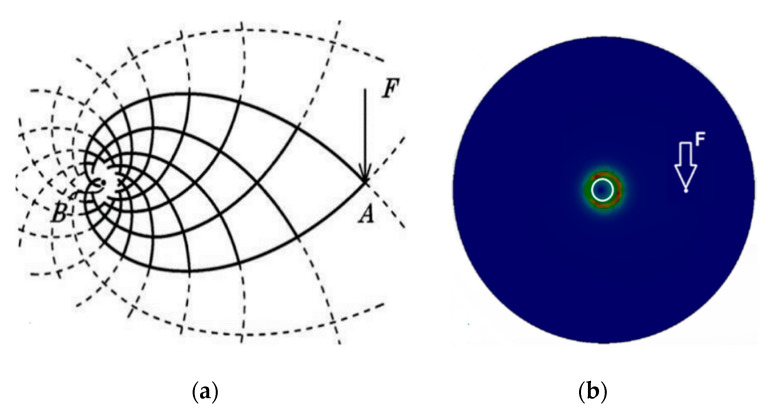
(**a**) The force F acting perpendicularly to the line joining the points AB (the lines indicate the principal stress directions) [4] and (**b**) The actual stress distribution from inserting the fixed circle of the continuum, simulated in the 3D program.

Under symmetrical load, the shape of the optimal truss, based on the Michell structure, is as follows (see Figure 2).

The individual dashed lines represent directions of the principal stresses during continuum loading (Figure 2) (e.g., of a continuous plate).

**Figure 3:**
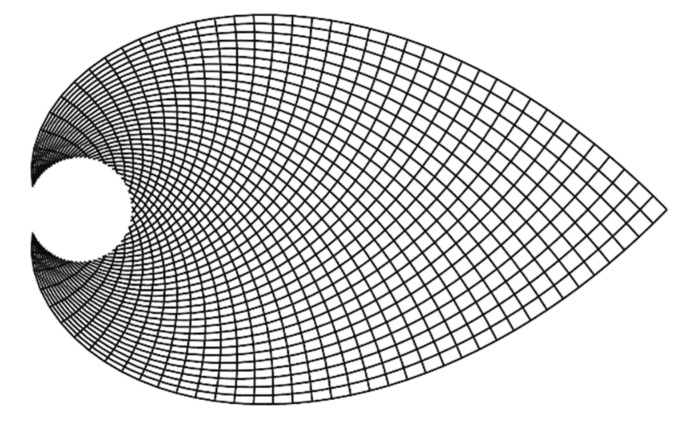
Dense mash solution in direction of principal stresses [8].

They are symmetrically distributed on the plate. The thick lines represent the ideal shape of the members that is fixed at point B and loaded at point A. The outline of the truss is determined by the principal stress lines between points A and B. The density of the principal stresses is highest at fixed point. Towards point A, the density of the principal stress lines decreases exponentially. Optimization can be performed by increasing the mesh density of members with the same cross-section (Figure 3).

**Figure 4:**
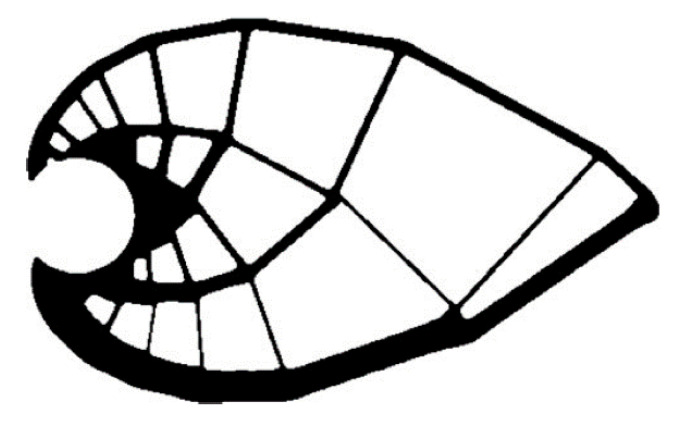
Gradual enlargement of the mash structure and simultaneous reduction in the member cross-section [9].

However, a different manner of achieving optimization is through a less dense mesh of members with a different cross-section (Figure 4). The mesh size increases geometrically from the fixed end.

The individual optimization methods do not determine the resulting degree of member density. There are methods in which the structure density is quite high, e.g., the narrow-band topology optimization method [10]. The Michel beam structure density depends on the choice of the step parameter [8].

The individual optimization methods do not necessarily form members in direction of the principal stresses. It follows from the optimization principle that the members are largely in the principal stress direction, but also often outside the principal stress direction. The paper [11] points out that it is most convenient to arrange the members in the principal stress direction with a reasonable member density.

**Figure 5:**
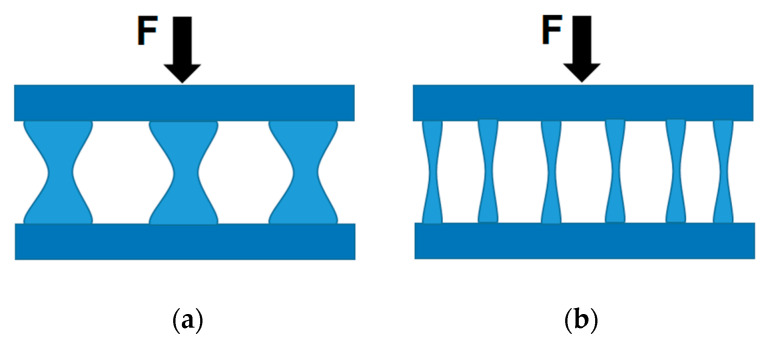
(**a**) Topology optimization with a smaller number of elements and (**b**) topology optimization with a larger number of elements.

The illustrative example shows two results of topology optimization (Figure 5). These results are equivalent because the weight, rigidity, and magnitude of the stresses in the elements are the same in both cases. Only the number of elements is different. The question is what condition would establish an adequate number of elements. The expected design of the number of elements forms the basis of the geometric optimization method proposed.

The process of changing the number of elements is also optimization and can be performed using a graphical method. The basis for the transfer of mass in the truss is to respect the principal stress directions. The members are formed in direction of the structure’s principal stresses.

## 2. Materials and Methods

The Michell truss was selected to examine the adequate number of elements. The following factors apply to cantilevered beams, determining the required density of structural elements.

**Figure 6:**
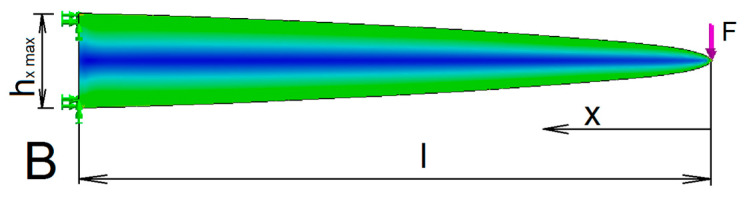
Load of the uniform stress cantilever of constant width (stress diagram).

The first factor is the shape that provides the same stress in the entire beam along the *x*-axis. With suitable weight reduction of the beam, a beam of the uniform stress can be obtained. In this case, the principal stress direction copies well the object’s contour (Figure 6). A disadvantage of such method of weight reduction are little material savings.

The cantilever of uniform stress meets the following condition for all cross-sections

(7) $$\sigma_{ox}=\sigma_{ox\max}$$

where $\sigma_{ox}$ is bending stress in direction of the *x* axis.

Based on this, the contour shape will correspond to a parabola

(8) $$h_{x}=h_{x\max}\sqrt{\frac{x}{l}}$$

where $h_{x}$ is the cross-sectional height of the beam depending on the axis *x* and *l* is the beam length.

This shape factor has little effect on the density of the distribution of the structure elements. However, it lends support to the principle that the density of elements should increase in direction to the fixed end of truss.

**Figure 7:**
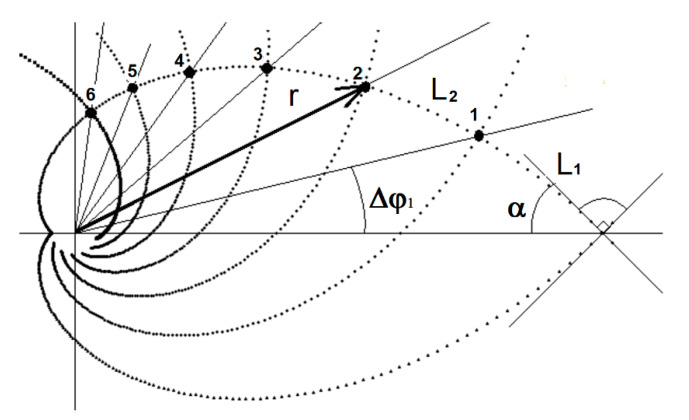
Orthogonal logarithmic spiral divided by gold section (*k* = 1, Δ*φ*_1_ = 27.5° and *α* = 45°).

The second factor is the shape of the principal stresses in the Michell truss. This shape results from the optimization condition (2). At first glance, it is clear that this is a mathematical function. The logarithmic spiral aligned perfectly with the principal stress direction. The question is what the parameters of this spiral are so that the curvature corresponds to the shape of the Michell truss. Only one parameter k=1 of the logarithmic curve satisfies the orthogonality condition. This is expressed by the following equation:

(9) $$r=e^{k\varphi }$$

where *r* is the radius of curvature of the logarithmic spiral, φ is the angle of rotation, and k is the polar slope (Figure 7).

In the Michell structure, the division density is formed by spirals of the opposite curvature direction, with the angle of rotation (Δφ) between the individual spirals being the same (Figure 7). The division of the spacings occurs at the intersection of the opposite curved logarithmic spirals. The size of the angle of rotation determines the degree to which the structure density increases. At a constant angle of rotation, the division is geometrical. The geometric quotient is given by the ratio of successive length sections of the beam division *L*_1_ and *L*_2_ (Figure 7), Equation (14). The increase in the structure’s density occurs in direction of the beam bindings. This can also be seen in Figure 3. This result also corresponds well with the first factor.

At the 27.5° angle of rotation, the geometric order quotient equals exactly to the golden section ratio. The same ratio is also maintained in this angle’s integer multiples (10). Since this is a natural principle, an ideal division of the member density can be assumed.

(10) $$\Delta \varphi_{i}=27.5i\;i=1,2,3,\ldots$$

Integer products no longer manifest the golden section ratio. With products, the structure becomes considerably dense. The length of the section on the logarithmic spiral is determined according to Equation (11).

(11) $$L(\varphi_{1},\varphi_{2})=\frac{r(\varphi_{2})-r(\varphi_{1})}{\sin\alpha }$$

(12) $$\Delta \varphi =\varphi_{2}-\varphi_{1}$$

(13) $$\sin\alpha =\frac{k}{\sqrt{k^{2}+1}}$$

(14) $$q=\frac{L_{2}(\varphi_{2},\varphi_{3})}{L_{1}(\varphi_{1},\varphi_{2})}=\frac{r(\varphi_{3})-r(\varphi_{2})}{r(\varphi_{2})-r(\varphi_{1})}$$

where $L(\varphi_{1},\varphi_{2})$ is the length of the section, q is the geometric quotient, and α is the polar slope angle.

The above equations are a tool for creating the Michell truss of different structural density. The trusses created in this way may be compared through optimization criteria. These criteria are based on achieving minimum weight and minimum compliance. Weight and compliance are the two parameters pointing out to the fact that the optimization has been successful. Only one monitored parameter can be created as a product of both of these parameters. The new parameter’s drawback is that information about the ratio between the weight and compliance parameter is lost. The advantage is a simple comprehensive assessment of the proposed trusses. The minimization condition is as follows

(15) $$\frac{m}{k}=\min$$

where m is the weight of the truss and k is the stiffness of the truss.

If a substantially flat truss is formed, where the surface dimensions significantly predominate over its thickness, then the area will represent the weight with an acceptable accuracy. Similarly, if the trusses are loaded with the same force at the same distance from the kinematics joints, the truss deflection at the free end will correspond to its compliance.

The inverse value will correspond to its stiffness. Of course, the same material is used for all beams, so the effect of the material does not have to be addressed.

The goal of optimization is then to achieve a minimum value of the product of area and deflection. Both parameters monitored should be as small as possible. Thus, a common parameter *Q* is obtained.

(16) Q=A.d

where A is the truss area and d the truss deflection.

It is recommended to choose a relative method for comparing the trusses. The relative advantage of the *Q* parameter is calculated from the percentage comparison (17).

(17) $$Q_{\%}=\frac{Q_{1}-Q_{2}}{Q_{1}}100$$

where $Q_{1}$ is the worse parameter and $Q_{2}$ is the better parameter of topology optimization.

The procedure for creating Michell trusses respects the following rules of the geometric method:

- The truss members may only be placed in the principal stress direction;
- The increase in the number of members is gradual;
- The location of the member must show the maximum possible improvement in the *Q* parameter.

## 3. Methodology

**Figure 8:**
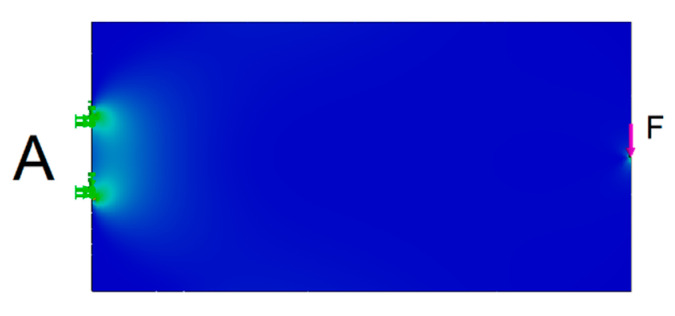
Load and joint of the base beam A (stress diagram).

Subject to detailed investigation was the optimization of the cantilevered beam. The basic beam A of rectangular shape (Figure 8) had the worst parameter in terms of weight optimization. It had the best values in terms of stiffness. Beams optimized in terms of stiffness and weight were compared with this basic beam. The position of the loading force and the outer kinematics joints of the truss were maintained for each truss.

The optimization was based on the shape of the ideal Michell truss. This truss was geometrically optimized in terms of member layout and density. The properties monitored were weight and stiffness. Individual forms of new trusses were assessed on the basis of the following principles of shape modifications.

1. Finding the optimal internal support of the Michell truss (Section 4.1)
2. Comparison of the effect of truss slenderness (Section 4.3)
3. Comparison of Michell truss modified into a leaf structure. This natural pattern occurred often, and a good optimization result was expected. (Section 4.4)

These three directions of modifications are subject to verification by graphical optimization. The results and conclusions resulting from the verification of the basic parameters are clarified in the following section.

In topology optimization, the golden section ratio was used only in selecting the parameters crucial for individual optimization iterations. Selection technology allows iteration acceleration while maintaining the decision sensitivity of evolutionary algorithms. Publications [12,13,14,15,16] deal with this in more detail.

## 4. Modelling and Results

The task of the experimental computation is to create an ideal truss based on certain principles. These principles form the basis of the proposed method of geometric optimization.

Experimental verification was performed with the SolidWorks (Dassault Systèmes SolidWorks Corporation, Waltham, MA, USA) calculation program. Parameters used in the SolidWorks 2018 Simulation module were material: alloy steel; solver type: FFE Plus; force: force—selected direction; and mesh type: solid mesh (tetrahedral elements). Adherence to a uniform maximum stress for all trusses was only approximate in this graphical method of designing the shape but, at the same time, it was sufficient to clearly determine the advantages and disadvantages of the truss structures compared.

The effort in case of all beams and trusses was to maintain the maximum stress (${\bar{\sigma }}_{T}$ = ${\bar{\sigma }}_{C}$=4.4 MPa) that occurred when the base beam was loaded (Figure 8).

**Figure 9:**
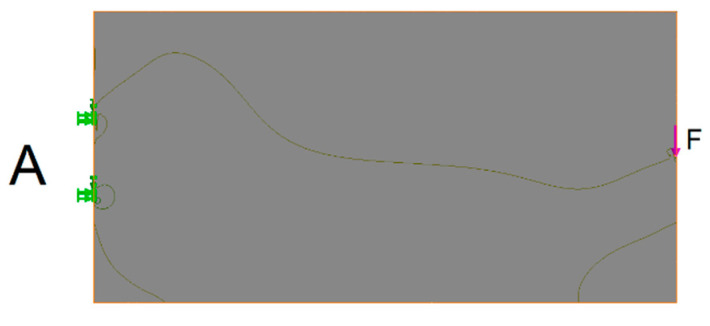
Direction of the principal compressive stresses in the basic beam.

The principal stress direction follows the contours of the object relatively slightly (Figure 9).

**Figure 10:**
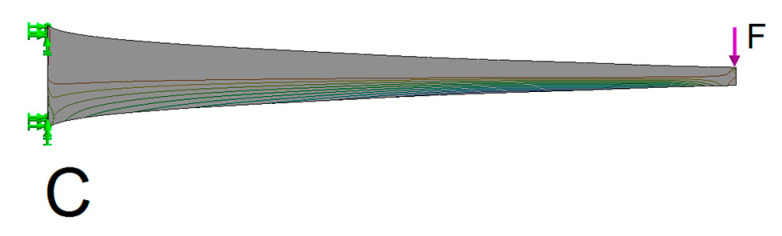
Direction of the principal compressive stresses of a parabolically formed beam (beam C) with distributed stress.

A slender beam with distributed stress C is obtained through the inverse shape of the beam of uniform stress (beam C, Figure 10). The advantage of this beam is that the maximum stress is distributed along the entire length of the beam, which reduces its weight. A disadvantage is that, at the same time, the stiffness of the beam is significantly reduced.

**Figure 11:**
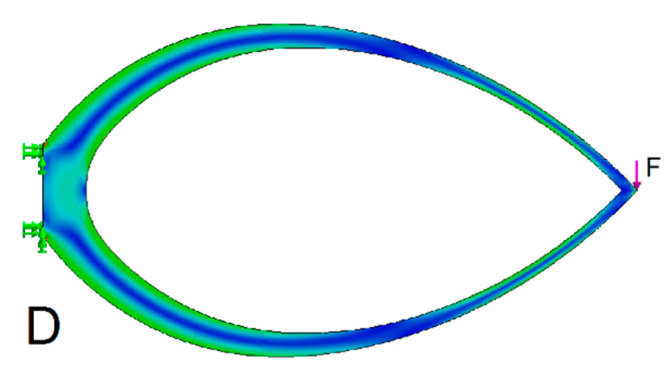
Stress diagram of a simple Michell truss D.

The simplest version of the Michell truss has only two arms. The arms are in the shape of the logarithmic spirals (truss D, Figure 11). The addition of inner members optimizes the resulting weight to stiffness ratio (the *Q* parameter). If the beam is sufficiently thin, the total weight of the beam will be represented by its side surface. Similarly, the overall stiffness of the beam will be represented by the deflection at the point and in the direction of the force load. All beams and trusses are dimensioned for the maximum stress present in the basic A beam (Table 1).

### 4.1. Determination of an Ideal Internal Support for Michell Truss D

**Figure 12:**
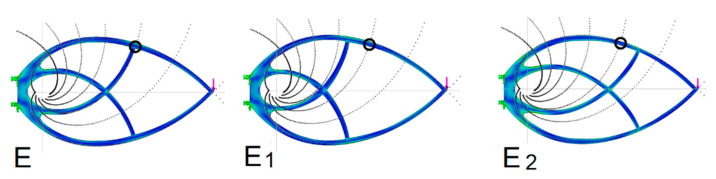
Determining the optimal support location.

The first comparison is based on determination of the optimal support point of the outer arch of the Michell truss (Figure 12), (Table 2). This point is obtained by retaining the second support curve (Figure 7). In this case, there will be only one support member under the arch (Figure 12, E truss). This truss was compared to trusses with the support point shifted in one and the other direction (the E1 and E2 trusses, respectively).

With a relatively small shift of the support point, a relative deterioration of more than 10% occurs in the *Q* parameter. This is a proof that the support established according to the golden section ratio yields the best stiffness results. The upper and lower arcs of the logarithmic spiral are divided by the support point in the golden section ratio (Figure 7). The result of an ideal support location is independent of knowing the golden section ratio. It is a natural principle that has determined the ideal location in accordance with the experimental modeling results.

### 4.2. Establishing the Appropriate Density of Internal Supports for Michell Truss

**Figure 13:**
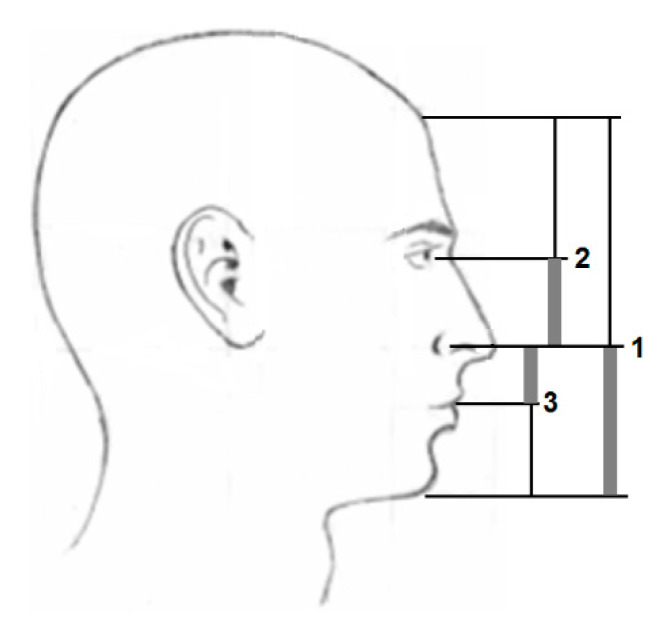
Face proportionality divided in the golden section.

The addition of further internal supports increases the truss stiffness, which improves the *Q* parameter. In this case, too, the natural principle was chosen when selecting the supports. The individual nodes respect the proportionality that is commonly found in natural patterns. Figure 13 shows, e.g., the division of the proportions of the face in the golden section with an alternating distribution of ratios.

**Figure 14:**
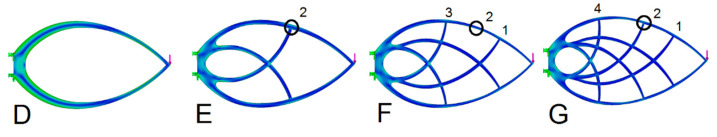
Increasing the number of internal support members of the Michell truss.

The improvement of the *Q* parameter depends not only on the number of internal support members but also on the method of their mutual spacings. The second comparison is based on the number of support members distributed evenly (Figure 14, Table 3). In this case, too, the distribution of new supports is spread around the optimal support point (see the E truss). Support curves 1 and 3 are retained for the two support points. Curves 1, 2, 4 are retained for the three support points (Figure 7). The support members are added gradually (Figure 14). The support points are selected at points with geometric growth, while the requirement of mass distribution according to the cantilever of uniform stress is also respected. Therefore, e.g., in the case of three supports, the points 1, 2, 4 are selected instead of points 1, 2, 3. In this way, a better *Q* parameter can be achieved.

**Figure 15:**
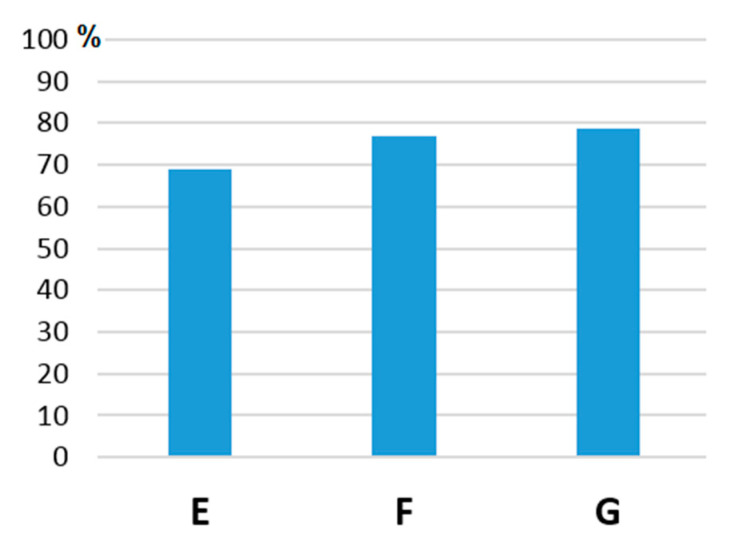
Rate of improvement through the increase in the number of inner members.

As you can see, adding one support rod improves the *Q* parameter by up to 69%. Further addition results in a less dramatic improvement (Figure 15).

The *Q* parameter improvement graph improves regressively with the addition of new members. Based on this, the third increase in density can be considered to be the optimal solution. Further increase has no longer a much greater effect, only the elements are getting thinner. Reduced element thickness leads to loss of truss stability.

### 4.3. Influence of the Truss’s Degree of Slenderness

**Figure 16:**
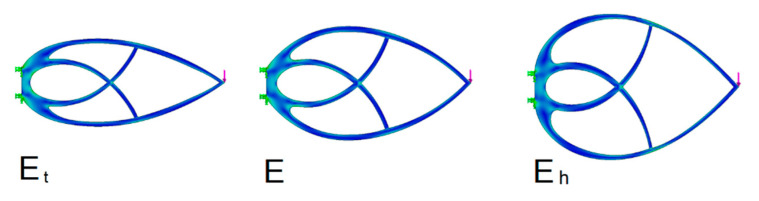
Trusses with a varying *k* parameter.

Orthogonality is violated during slenderness reduction, but the results are better (Figure 16 and Table 4). There is a certain optimum for reducing the Michell truss slenderness.

According to the results, the most convenient slimmed down shape is 17% better than the orthogonal shape. The physical cause of this phenomenon is that the rods are able to transfer the bending stress to some extent, too. The optimization principle used is based on a graphical estimate of the truss shape, which introduces a certain uncertainty caused by human factors into the measured results.

### 4.4. Combination of a Michell Truss and the Beam C

**Figure 17:**
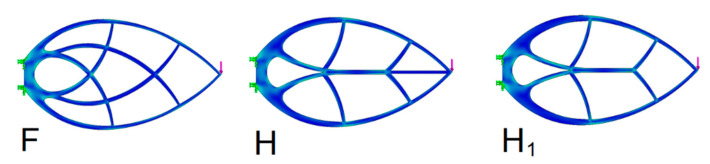
Michell truss F and the orthogonal leaf truss H.

Unlike the slender beam C, the Michell truss does not have a central structure. As can be seen, the material savings can also be achieved through the beam C, which is also a pattern often occurring in nature, e.g., tree branches or herb stems. The combination of these principles also occurs in the form of a leaf structure. In technical practice, these leaf structures are not yet consciously used to the fullest. In this respect, it is appropriate to check the leaf beam against the Michell truss of a similar structure. The optimization parameters were checked in trusses with two internal support members. The shape of trusses with orthogonal structure was verified (Figure 17 and Table 5).

As can be seen, the H truss deteriorated by 18% compared to the F truss. However, the H truss improved by approximately 55% compared to the original C beam.

## 5. A Note on Natural Patterns

Increasing the number of elements in topology optimization usually creates shapes that resemble natural patterns. The result of topology optimization is that biomorphic shapes are formed that show the best stiffness to weight ratio. This technical indicator is similar in nature to the complex indicator used in the economy in the form of the product performance to price ratio. The following works deal with structures that often resemble natural shapes [17,18,19,20].

**Figure 18:**
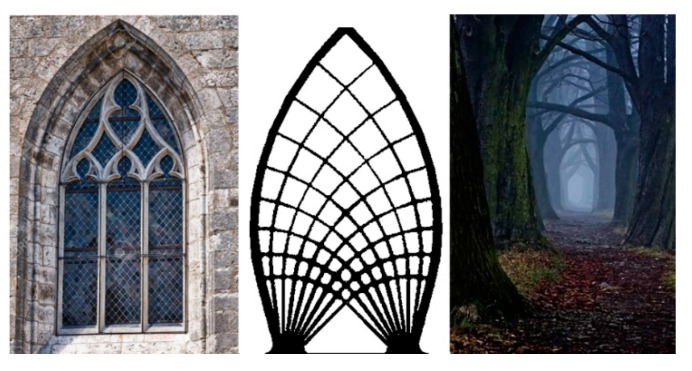
Gothic vaults in the shape of the Michell’s ideal truss contour and the nature-inspired interpretation.

**Figure 19:**
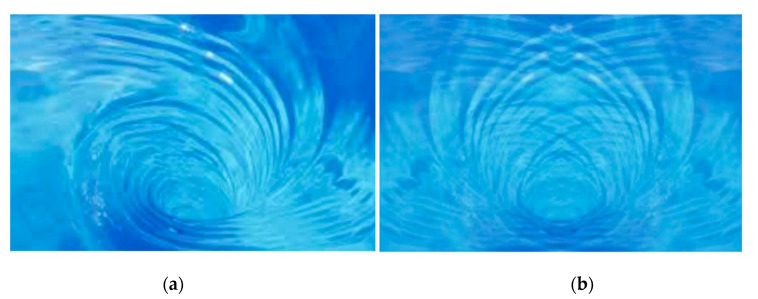
(**a**) The shape of streamlines in the vortex. (**b**) The mirror image laid over the original one creates the shape of the Michell beam.

The ideal biomorphic shape of the structure shows good agreement with the shape of the principal stress propagation [21]. This knowledge may also be used in the intuitive design of a suitable structure shape. Evidence of this is, e.g., the ideal vaults of the Gothic style. The designs were created at a time when there were no modern computational schemes or computers (see Figure 18). Of course, there are different directions of loads in these objects, but in principle, shapes similar to a logarithmic spiral are created, e.g., tree branching (Figure 18). Even ideal vortex streamlines are subject to this principle (Figure 19).

**Figure 20:**
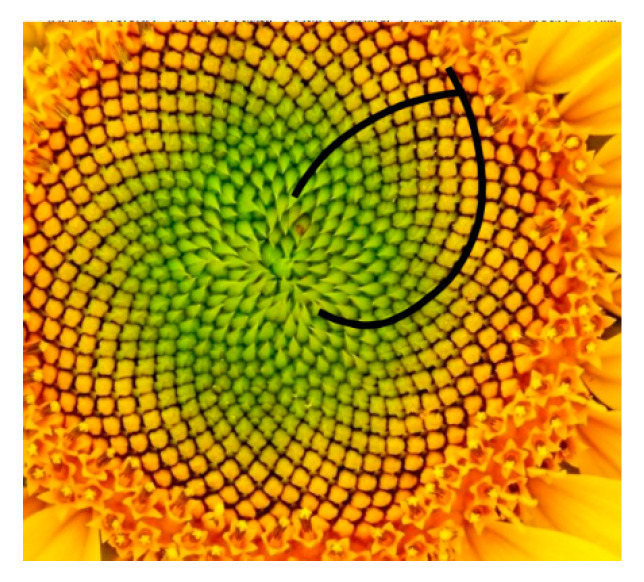
Spirals in the sunflower.

Natural patterns preserve both the orthogonality and the golden section, but the way in which they do so differs. For example, the sunflower has seeds arranged in quasi-logarithmic spirals (they are not mathematically accurate logarithmic spirals). However, the manner in which the opposite spirals connect is always orthogonal (Figure 20). The ratio of the number of spirals in the clockwise direction to the number in the counterclockwise direction is the ratio of two adjacent Fibonacci numbers, 21 and 54. This ratio of numbers always approaches the golden section ratio. However, this is not a loaded beam.

## 6. Discussion

Natural patterns are undoubtedly the ones best optimized. The Michell truss is a natural shape that is created through the application the principles of nature. One such principle is to divide the length of the logarithmic spiral in a geometric order by the quotient of the golden section ratio. It is this principle that ensures not only the ideal ratio of weight and stiffness but also the ideal member density. This member division cannot be, however, obtained from the known optimization conditions. The result was brought about by the geometric method based on copying the proportionality principles.

From the simulation, it was also found that the addition of new support members improves the *Q* parameter only slightly. Although the addition of new support members leads to a slight improvement in the *Q* parameter, the cross-section of the elements becomes thinner in the process. Such reduction in slenderness reduces stability to such degree that the structure may collapse. The ideal increase in the member density is only to the second degree. Structural instability and volumetric topology optimization have been discussed in more detail in the study [11].

The Michell truss represents an ideal condition without design restrictions. It achieves maximum stiffness at minimum weight. In case of design restrictions, the result is worse, as shown in Section 4. Restrictions of this type are discussed in the paper [22].

It also seems that if the truss members lack revolute joint, then the rods can also be loaded to a certain extent by bending. Such load arises in a nonorthogonal structure. Nonorthogonality is obtained by slenderness the ideal Michell truss. The result is an even better weight and stiffness parameter.

The leaf-shaped trusses do not exceed the ideal Michell truss parameters. Overall, the parameters are good in terms of shape optimization. This structure can have advantages in terms of production technology or the purpose of use. For example, buildings are often of a vertical structure with traffic roads running alongside.

**Figure 21:**
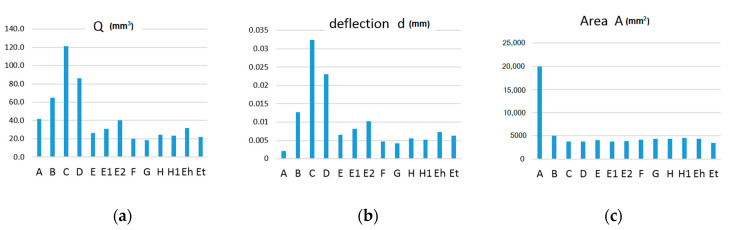
Comparison of shapes of individual beam and truss graphs of: (**a**) *Q*, (**b**) *d* and (**c**) *A* parameters.

The assessment of the topology optimization success according to the *Q* parameter has its pitfalls. The *Q* parameter points to beam improvement only generally, leaving the ratio of the contribution of weight and stiffness unclear. In the investigated cases, the dominant component was the stiffness or compliance. By comparing the beam surfaces and deflection with the corresponding *Q* parameter, it can be seen that the relative shape of the deflection graph shows only small differences from the *Q* parameter graph (Figure 21).

## 7. Conclusions

The proposed methodology of geometric topology optimization can be used to build an optimization program that will accept geometric optimization. This means that the internal structure of the trusses is optimized by creating support points in the principal stress direction and these support points are divided by the golden section ratio. The presented methodology of geometric optimization can theoretically be applied to all the optimization algorithms known. For example, the truss-based optimization methods, voxel-based methods, ground structure method, SIMP (Solid Isotropic Material with Penalisation) technique, FE (Finite Element) analysis technique, and the like.

Truss optimization saves materials. Additive technologies that can produce optimized structures are already intensively reducing the cost of energy and construction materials used, which is a major benefit for environmental protection and conservation of natural resources.

## Figures and Tables

**Table 1:** Comparison of the B, C beams, and D truss to the basic A beam.

Beam, Truss	Max Stress (MPa)	*A* Area (mm^2^)	*d* Bend (mm)	*Q* (mm^3^)	*Q*_%_	Meaning
A	4.31	20,000	0.00208	41.6	–	
B	4.17	5073	0.012771	64.8	36	Deterioration
C	4.14	3732	0.032478	121.2	66	Deterioration
D	4.34	3731	0.02306	86.0	52	Deterioration

**Table 2:** Comparing the influence of the inner member support point in a Michell truss.

Truss	Max Stress (MPa)	*A* Area (mm^2^)	*d* Bend (mm)	*Q* (mm^3^)	*Q*_%_	Meaning
E	4.27	4031	0.006592	26.6	–	
E1	4.46	3762	0.008197	30.8	14	Deterioration
E2	4.37	3902	0.010281	40.1	34	Deterioration

**Table 3:** Comparing the influence of the of the number of members in the Michell truss.

Truss	Max Stress (MPa)	*A* Area (mm^2^)	*d* Bend (mm)	*Q* (mm^3^)	*Q*_%_	Meaning
D	4.34	3731	0.02306	86.0	–	
E	4.27	4031	0.006592	26.6	69	Improvement
F	4.34	4165	0.004771	19.9	77	Improvement
G	4.44	4376	0.004213	18.4	4.44	Improvement

**Table 4:** Comparison of trusses of varying slenderness with one support.

Truss	Max Stress (MPa)	*A* Area (mm^2^)	*d* Bend (mm)	*Q* (mm^3^)	*Q*_%_	Meaning	*k*
E_t_	4.37	3502	0.006334	22.2	–		0.3
E	4.27	4031	0.006592	26.6	17	Deterioration	1
E_h_	4.36	4357	0.007249	31.6	30	Deterioration	0.7

**Table 5:** Comparison of the influence of the combination of the beam C and the Michell truss F.

Truss Beam	Max Stress (MPa)	*A* Area (mm^2^)	*d* Bend (mm)	*Q* (mm^3^)	*Q*_%_	Meaning
F	4.34	4165	0.004771	19.9	–	
H	4.29	4341	0.005576	24.2	18	Deterioration
H1	4.31	4504	0.005234	23.6	16	Deterioration
C	4.14	3732	0.032478	121.2	84	Deterioration
